# Hyperbranched polyglycerol is superior to glucose for long-term preservation of peritoneal membrane in a rat model of chronic peritoneal dialysis

**DOI:** 10.1186/s12967-016-1098-z

**Published:** 2016-12-13

**Authors:** Caigan Du, Asher A. Mendelson, Qiunong Guan, Ghida Dairi, Irina Chafeeva, Gerald da Roza, Jayachandran N. Kizhakkedathu

**Affiliations:** 1 Department of Urologic Sciences, University of British Columbia, Vancouver, BC Canada; 2 Division of Nephrology, Department of Medicine, University of British Columbia, Vancouver, BC Canada; 3 Department of Pathology and Laboratory Medicine, Centre for Blood Research, University of British Columbia, Vancouver, BC Canada; 4 Department of Chemistry, University of British Columbia, Vancouver, BC Canada; 5 Jack Bell Research Centre, 2660 Oak Street, Vancouver, BC V6H 3Z6 Canada; 6 London Health Sciences Centre, London, ON Canada

**Keywords:** PD solution, Biocompatibility, Hyperbranched polyglycerol, Long-term PD, Peritoneal membrane

## Abstract

**Background:**

Replacing glucose with a better biocompatible osmotic agent in peritoneal dialysis (PD) solutions is needed in PD clinic. We previously demonstrated the potential of hyperbranched polyglycerol (HPG) as a replacement for glucose. This study further investigated the long-term effects of chronic exposure to HPG as compared to a glucose-based conventional PD solution on peritoneal membrane (PM) structure and function in rats.

**Methods:**

Adult male Wistar rats received once-daily intraperitoneal injection of 10 mL of HPG solution (1 kDa, HPG 6%) compared to Physioneal™ 40 (PYS, glucose 2.27%) or electrolyte solution (Control) for 3 months. The overall health conditions were determined by blood chemistry analysis. The PM function was determined by ultrafiltration, and its injury by histological and transcriptome-based pathway analyses.

**Results:**

Here, we showed that there was no difference in the blood chemistry between rats receiving the HPG and the Control, while PYS increased serum alkaline phosphatase, globulin and creatinine and decreased serum albumin. Unlike PYS, HPG did not significantly attenuate PM function, which was associated with smaller change in both the structure and the angiogenesis of the PM and less cells expressing vascular endothelial growth factor, α-smooth muscle actin and MAC387 (macrophage marker). The pathway analysis revealed that there were more inflammatory signaling pathways functioning in the PM of PYS group than those of HPG or Control, which included the signaling for cytokine production in both macrophages and T cells, interleukin (IL)-6, IL-10, Toll-like receptors, triggering receptor expressed on myeloid cells 1 and high mobility group box 1.

**Conclusions:**

The results from this experimental study indicate the superiority of HPG to glucose in the preservation of the peritoneum function and structure during the long-term PD treatment, suggesting the potential of HPG as a novel osmotic agent for PD.

**Electronic supplementary material:**

The online version of this article (doi:10.1186/s12967-016-1098-z) contains supplementary material, which is available to authorized users.

## Background

Peritoneal dialysis (PD) is one of the effective options for treating renal failure in patients in nephrology clinics, evidenced by the fact that as compared to hemodialysis (HD), PD has better or equal clinical outcomes [1–3], and is also associated with an increase in quality-of-life and therapy-satisfaction scores in patients [4, 5]. Dianeal and Physioneal (PYS) from Baxter are the most common solutions for PD, and contain a high concentration of glucose as an osmotic agent. However, increasing evidence in the literature clearly demonstrates that the long-term exposure to these glucose-containing PD solutions is associated with systemic health complications (e.g. an increase in cardiovascular disease and the loss of residual kidney function) in PD patients particularly for those with diabetes [6–10], and causes local deleterious effect on the peritoneum—leading to ultrafiltration failure (UFF) and resulting in poor outcome of PD [11, 12]. Thus, there is an unmet need for glucose-free PD solutions in order to improve clinical outcomes of PD.

Hyperbranched polyglycerol (HPG) is a highly hydrophilic, water-soluble branched polyether polymer, and can be prepared by a one-step synthesis via ring-opening multi-branching polymerization of glycidol [13]. Recent reports in literature demonstrate the biocompatibility and potential use of this macromolecule in many different biomedical applications, such as a human serum albumin substitute [14], a drug carrier [15, 16] and a colloid for cold preservation of cells or organs [17]. Our preliminary studies have shown that HPG (0.5–3 kDa)-based PD solutions can be prepared by varying the HPG concentration from 2.5 to 15% (w/v) within an osmolality range (294–424 mOsm/kg) and neutral pH (6.6–7.4) that are comparable to Dianeal (2.5% glucose, 395 mOsm/L, pH 5.2) and PYS (2.27% glucose, 395 mOsm/L, pH 7.4) [18, 19], and by a single dwell time these HPG-based PD solutions produced similar or significantly better fluid and waste removal while causing less damage to peritoneal membrane (PM) in rats compared to either Dianeal (2.5% glucose) or PYS (2.27% glucose) [18, 19]. The objective of the current study was to investigate the long-term effects of HPG-based PD solution (denoted here as HPG) compared to conventional glucose-based PYS in rats, especially on the PM structure and function.

## Methods

### Reagents and animals

Physioneal™ 40 solution (denoted as PYS here) was purchased from Baxter Healthcare Co. (Deerfield, IL, USA). Primary antibodies were: mouse anti-vascular endothelial growth factor (VEGF) antibody (Cat#: NB100-664, Novus Biologicals, Littleton CO, USA), mouse monoclonal anti-α-smooth muscle actin (SMA) (clone 1A4, Sigma-Aldrich, Oakville, ON, Canada), and mouse monoclonal anti-MAC387 antibody (Clone MAC387, Santa Cruz Biotech Inc., Dallas, TX, USA), and mouse monoclonal anti-β-actin (clone AC-40, Sigma-Aldrich, Canada). Secondary horseradish peroxidase (HRP)-conjugated anti-mouse IgG HRP (sc-2314) was from Santa Cruz Biotech.

Male outbred Wistar rats (~400 g bodyweight, 12–14 weeks old) were purchased from the Charles River Laboratories International, Inc. (Wilmington, MA, USA), and maintained in the animal facility of the Jack Bell Research Centre at the University of British Columbia (UBC) (Vancouver, BC, Canada). Animal experiments were performed in accordance with the Canadian Council on Animal Care guidelines under protocols approved by the animal use subcommittee of UBC.

### Synthesis and characterization of HPG

HPG (1 kDa) was synthesized by anionic ring opening multi-branching polymerization, and its characteristics were verified as described previously [18, 19].

### Preparation of HPG-based and control solutions

HPG solution (denoted as HPG, approximately 402 mOsmol/kg, pH 7.4) was prepared by dissolving of HPG (1 kDa, 6% w/v) in PD buffer solution similar to PYS solution containing sodium chloride (NaCl, 538 mg/100 mL), sodium lactate (NaC_3_H_5_O_3_, 168 mg/100 mL), calcium chloride dehydrate (CaCl_2_ 2H_2_O, 18.4 mg/100 mL), magnesium chloride hexahydrate (MgCl_2_ 6H_2_O, 5.1 mg/100 mL) and sodium bicarbonate (NaHCO_3_, 210 mg/100 mL). The osmolality and pH of HPG solution were approximately the same as those of PYS (2.27% glucose, 395 mOsm/L, pH 7.4). The electrolye solution (denoted as Control, 251 mOsmol/kg, pH 7.4) had the same composition as HPG solution except that the HPG polymer was omitted. Both HPG and the electrolyte solutions were filter-sterilized prior to use in rats.

### Chronic rat model of PD, bodyweight and peritoneal equilibrium test (PET)

Chronic rat model of PD—the Wistar rats receiving once-daily intraperitoneal instillation of 10 mL of a PD solution was established as previously described [20–22]. In brief, after anesthesia with isoflurane, 10 mL of a pre-warmed PD solution (HPG, PYS or Control) were slowly injected to the peritoneal cavity at the abdominal midline. Animals awoke within 1–2 min after the procedure, and had free access to food and tap water. Animals in Sham group were treated in the same manner including the needle puncture but without fluid injection.

After 3 months of daily exposure to the PD solutions, both bodyweight gain (BWG) and PM ultrafiltration (UF) were determined. BWG in each rat was determined as follows: BWG (%) = (final bodyweight—initial bodyweight)/initial bodyweight. The PM function was examined using UF. In brief, the UF was performed by intraperitoneal injection (IP) of 30 mL of PYS. After 4 h of dwelling time, the peritoneal fluid was recovered using a syringe, and its volume was used for calculating the UF capacity.

### Histological analyses

After UF measurement, two strips of parietal peritoneum from each rat were collected, one from left lumbar abdominal region and the other from the right region. The histological analysis of the submesothelial thickness was performed in hematoxylin and eosin (H&E) stained tissue section as described previously [18]. The number of the blood vessel and capillaries per millimeter of the peritoneum section was counted longitudinally in the PM (both submesothelial and muscle-associated connective tissue layers) of the whole tissue sections. In addition to H&E stain, the tissue sections were also stained with Masson’s trichrome method for the examination of collagen fibers.

### Immunohistochemical analysis

The expression of VEGF, α-SMA and MAC387 in the tissue sections of six rats randomly selected from each group was examined using a routine immunohistochemical method as described by our lab [23].

### Tissue preparation and RNA extraction

Four rats were randomly selected from each group except of three in Sham. The parietal peritoneum tissues were collected as described in above “Histological Analyses” section, and were snap-frozen with liquid nitrogen first, and then stored in −80 °C until use. Total RNA was extracted by using mirVana™ isolation kit (Ambion, Austin, TX, USA) from the PM that was carefully pealed off from the frozen peritoneum tissue. The RNA samples with ≥8 of RNA Integrity Number (RIN) were selected for microarray analysis.

### Microarray and ingenuity pathway analyses (IPA)

Microarray analysis was performed in the Laboratory for Advanced Genome Analysis at Vancouver Prostate Centre (Vancouver, BC, Canada) following a standardized protocol as described previously [24]. Only the genes with *p* ≤ 0.05 (t test) and ≥ twofold change were uploaded into IPA software (Ingenuity Systems, Redwood City, CA, USA) to examine gene enrichment pathways.

The signaling pathway search was performed using the keywords related to inflammation, such as “B cell development”, “complement”, “granulocyte/agranulocyte adhesion and diapedesis”, “antigen presentation pathway”, and so on, to identify all the genes related to these cellular functions. After that, the genes associated with these pathways were separately imported into the “Canonical Pathway” frame of the IPA software to determine whether or not the signaling pathways were significantly regulated by each treatment (HPG, PYS or Control) compared to Sham or HPG or PYS compared to Control.

### Blood chemistry

A comprehensive metabolic panel of 14 blood substances, including alanine aminotransferase (ALT), albumin (ALB), alkaline phosphatase (ALP), amylase (AMY) total calcium (Ca^2+^), creatinine (Cre), globulin (Glob), glucose, phosphorus (Phos), potassium (K^+^), sodium (Na^+^), total bilirubin (TBIL), total protein (TP), and urea nitrogen (BUN), in heparinized whole blood samples were determined in blood using the VetScan^®^ Comprehensive Diagnostic Profile reagent rotor with the VetScan Chemistry Analyzer VS2 (Abaxis, Inc., Union City, CA, USA) in the Animal Unit Hematology Diagnostic Laboratory at Jack Bell Research Centre (Vancouver, BC, Canada).

### Statistical analysis

Data were presented as mean ± standard derivation (SD) of each group. Both Student’s t test with two-tailed distribution and analysis of variance (ANOVA) (GraphPad Prism software, Inc., La Jolla, CA, USA) were used as appropriate for data analyses. A p value of ≤0.05 was considered significant.

## Results

### Unlike PYS, HPG solution does not have specific impact on overall health status

**Fig. 1:**
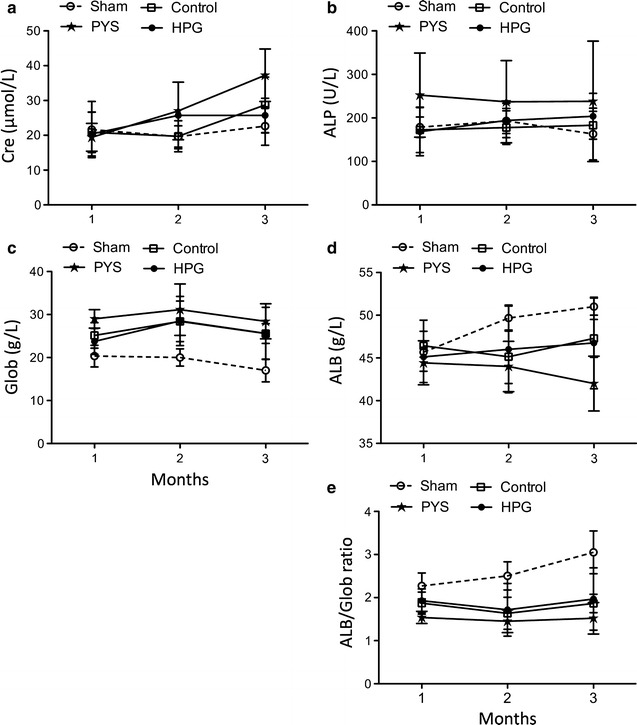
The chemical changes in the blood of rats after 3 months of intraperitoneal injection of PD solutions. Male Wistar rats received once-daily intraperitoneal injection of Control (n = 7), PYS (n = 7) or HPG (n = 8), or needle puncture as Sham Control (n = 3). The blood chemistry test was performed three times during 3-month period of treatment (once at the end of each month). **a** Creatinine (Cre) (PYS vs. Control: p = 0.0231, HPG vs. Control: p = 0.8439, HPG vs. PYS: p = 0.0269). **b** Alkaline phosphate (ALP) (PYS vs. Control: p = 0.0074, HPG vs. Control: p = 0.4325, HPG vs. PYS: p = 0.0204). **c** Globulin (Glob) (PYS vs. Control: p = 0.0141, HPG vs. Control: p = 0.7534, HPG vs. PYS: p = 0.0171). **d** Albumin (ALB) (PYS vs. Control: p = 0.003, HPG vs. Control: p = 0.7796, HPG vs. PYS: p = 0.0403). **e** ALB/Glob ratio (PYS vs. Control: p = 0.0022, HPG vs. Control: p = 0.5636, HPG vs. PYS: p = 0.0114). Data were presented as mean ± standard derivation (SD) of each group, and were statistically analyzed using two-way ANOVA

The overall health status of rats was determined by both BWG and changes in a comprehensive metabolic panel of 14 blood substances that represent the basic metabolism (glucose) and electrolytes (Na^+^, K^+^, Ca^2+^, and Phos), and markers of both kidney and liver functions. After 3 months of daily treatment with different solutions, there was no significant difference in the BWG in Control (46.04 ± 15.88%, n = 7), PYS (39.75 ± 15.66%, n = 7) and HPG (35.01 ± 5.95%, n = 8) as compared to Sham (50.67 ± 12.42%, n = 3) using one-way ANOVA with Dunnett’s multiple comparison test (p = 0.246). The blood chemistry test was performed once every month during 3 months of the treatment. Overall, the changes of the blood substances in response to daily injection of HPG solution versus electrolyte Control (without HPG) or Sham were not significantly different (Additional file 1: Table S1). As compared to the initial levels (prior to the treatment), the treatment procedure nonspecifically (regardless of Sham or injection of any of Control, PYS and HPG solutions) induced an increase in Na^+^, TBIL and TP, and a decrease in Phos (Additional file 1: Table S1). However, as compared to Control or HPG, IP injection of PYS significantly changed the levels of Cre, ALP, ALB and Glob (including ALB/Glob ratio) during 3 months of treatment (Sham: n = 3; Control: n = 7; PYS: n = 7; HPG: n = 8). As shown in Fig. 1 or in Additional file 1: Table S1, unlike HPG, PYS increased serum levels of Cre significantly in comparison to the Control or HPG (PYS vs. Control: p = 0.0231, HPG vs. Control: p = 0.8439, HPG vs. PYS: p = 0.0269, two-way ANOVA) (Fig. 1a), ALP (PYS vs. Control: p = 0.0074, HPG vs. Control: p = 0.4325, HPG vs. PYS: p = 0.0204, two-way ANOVA) (Fig. 1b) and of Glob (PYS vs. Control: p = 0.0141, HPG vs. Control: p = 0.7534, HPG vs. PYS: p = 0.0171, two-way ANOVA) (Fig. 1c). The levels of ALB in PYS but not HPG decreased as compared to that in Control (PYS vs. Control: p = 0.003, HPG vs. Control: p = 0.7796, HPG vs. PYS: p = 0.0403, two-way ANOVA) (Fig. 1d), and more significant differences were seen from the calculation of ALB/Glob ratio among these groups (PYS vs. Control: p = 0.0022, HPG vs. Control: p = 0.5636, HPG vs. PYS: p = 0.0114, two-way ANOVA) (Fig. 1e).

### Unlike PYS, HPG solution does not specifically attenuate PM function

**Fig. 2:**
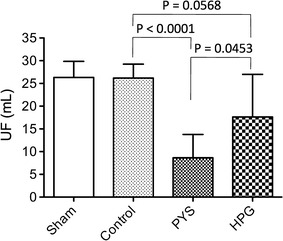
HPG has better preservation of peritoneal membrane function. The ultrafiltration as a parameter of peritoneal function was measured after 3 months of daily exposure of peritoneal cavity to the PD solutions. Each rat received intraperitoneal injection of 30 mL of PYS. After 4 h of dwell time, the fluid was recovered from the peritoneal cavity using a syringe, and its volume was measured. Data were presented as mean ± SD of each group (Sham: n = 3; Control: n = 7; PYS: n = 7; HPG: n = 8), and were statistically analyzed using two-tailed t test

The PM function was determined using UF capacity after 3 months of daily exposure to Control, PYS or HPG solution. As shown in Fig. 2, there was no significant difference in the UF of the PM between Sham (26.33 ± 3.51 mL, n = 3) and Control (26.17 ± 3.06 mL, n = 7). Statistical analysis using two-tailed t test indicated that as compared to Control group, no significant difference from HPG group was found in this limited number of animals (17.64 ± 9.34 mL, n = 8) (p = 0.0568), while the rats in PYS group lost the PM function (8.64 ± 5.14 mL, n = 7) as compared with Control (p < 0.0001) or HPG (p = 0.0453). After 4 h of dwell time only a few milliliters of dialysate were recovered in some rats, and were heavily “contaminated” with tissue debris and the blood, so that we were not able to determine the PM function using other parameters, such as the removal of the urea, creatinine, or sodium in these samples.

### Unlike PYS, HPG solution does not significantly induce morphological and structural changes of PM

**Fig. 3:**
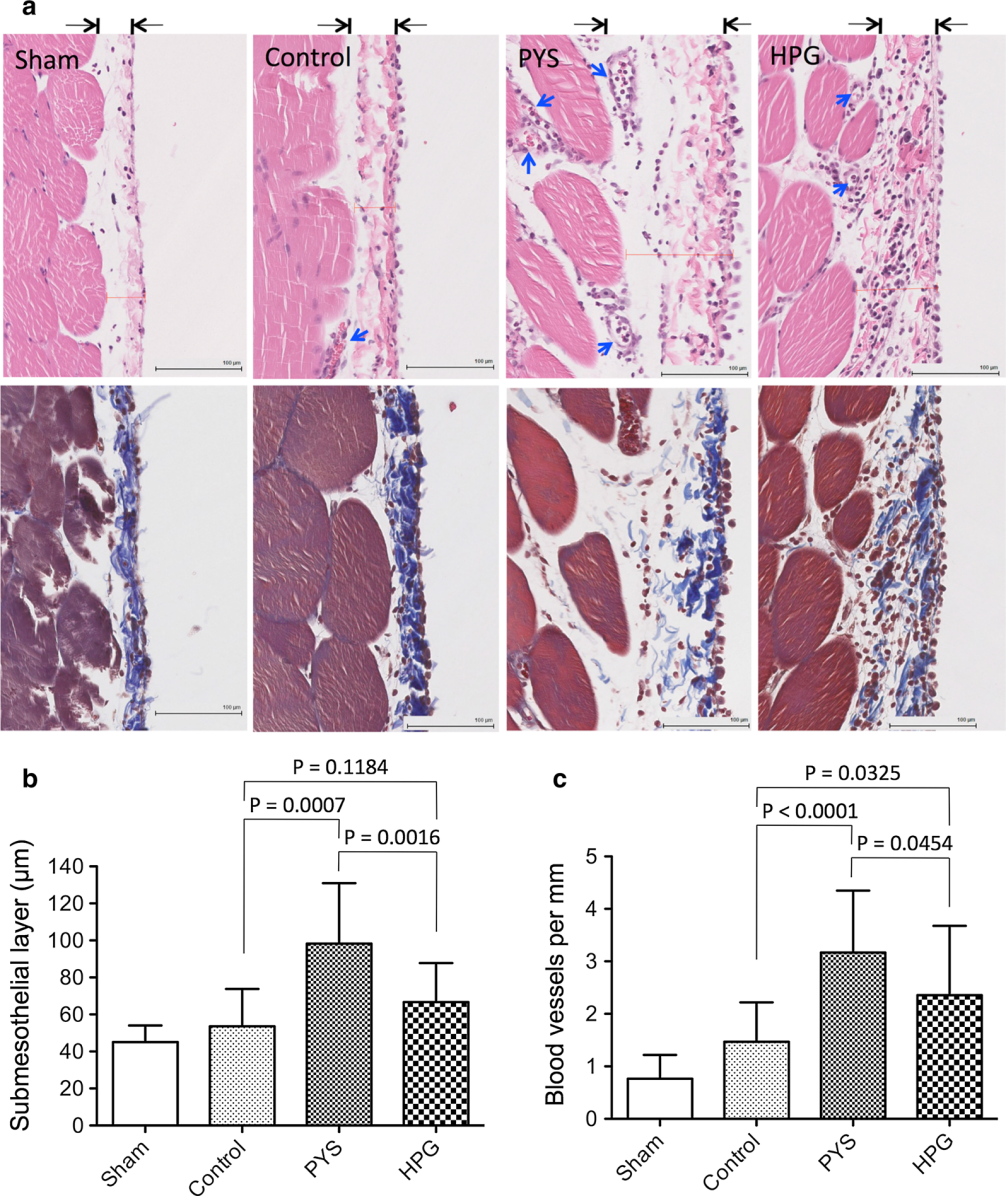
HPG has better protection of peritoneal membrane structure. At the end of 3-month treatment, peritoneal tissue sections (2 sections/rat) were taken from rats (Sham: n = 3; Control: n = 7; PYS: n = 7; HPG: n = 8) after 4 h of PYS exposure for UF test. **a** A typical microscopic image of the peritoneal tissue sections in each group, and were stained using either H&E (*top panel*) or Masson’s trichrome method (*bottom panel*). H&E stain showing the thickness of the peritoneal membrane indicated by the distance between *two arrows*. *Nuclear dark blue stain* cellular infiltrates; *blue arrows* blood vessels; *black thin bar* 100 µm. Masson’s trichrome stain showing collagen deposition (*dark blue*). **b** The thickness of submesothelial layer in each tissue section was measured using the Digital Image Hub software. Data were presented as mean ± SD of each group, and were statistically analyzed using two-tailed t test. **c** Angiogenesis in the peritoneal membrane was represented by the number of the blood vessels and capillaries per millimeter. Data were presented as mean ± SD of each group, and were statistically analyzed using two-tailed t test

To investigate the pathological changes in the PM after daily exposure to different solutions (particularly PYS and HPG), histology analysis of the PM was performed after UF determination. In H&E stained sections (Fig. 3a, top panel), the number of small blood vessels and capillaries in the deeper loose adipose connective tissues, and the thickness of the submesothelial compact zones were examined. As showed in Fig. 3b, rats in PYS group had the thickest submesothelial compact zones (98.25 ± 32.73 µm, n = 14 sections of seven rats), which were significantly larger than those in Control group (53.57 ± 20.2 µm, n = 14 sections of seven rats) (p = 0.0007, two-tailed t test) or in HPG group (66.68 ± 21.11 µm, n = 16 sections of eight rats) (p = 0.0016, two-tailed t test). Also, there was no statistical difference in the thickness of the submesothelial layer between the Control and HPG groups (p = 0.1184, two-tailed t test). Similar to the changes in the submesothelial layer thickness in these groups, more blood vessels and capillaries in PYS group (3.17 ± 1.18 per mm, n = 14 sections of seven rats) were seen than those of Control group (1.47 ± 0.75 per mm, n = 14 sections of seven rats) (p < 0.0001, two-tailed t test) or HPG group (2.36 ± 1.32 per mm, n = 16 sections of eight rats) (p = 0.0454) (Fig. 3c). Furthermore, the trichrome stain indicated the deposition of collagen (blue color) within the PM. As shown in Fig. 3a (bottom panel), there was clearly an increase in collagen accumulation in all of 3 groups (Control, PYS and HPG) as compared to that in Sham, but it was difficult to quantitatively compare the differences in the collagen accumulation between Control and PYS or HPG because the integrity of collagen deposition might be affected by the test of UF.

### Compared to PYS, HPG solution induces less VEGF expression, macrophage activation and α-SMA-expressing cell differentiation

**Fig. 4:**
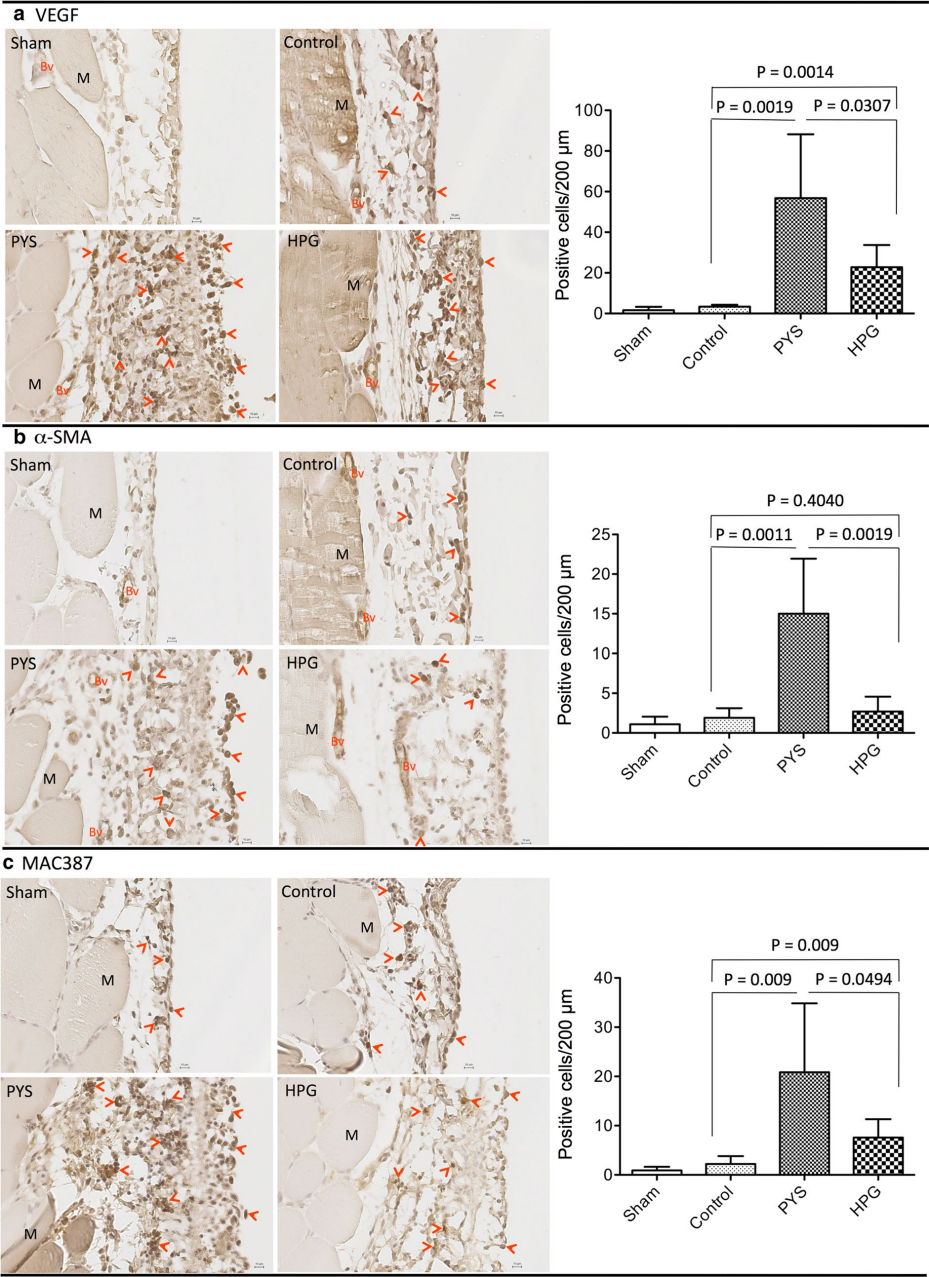
HPG induces less VEGF production, less myofibroblast differentiation and lower macrophage activation. The expression of VEGF, α-SMA and MAC387 in the peritoneal tissue sections was examined using a routine immunohistochemical method. Data were a typical microscopic view of the peritoneal tissue sections in each group. **a** VEGF was detected using mouse monoclonal anti-VEGF antibody from Novus. *Left graph* a typical microscopic view, *Dark brown stain* VEGF-expressing cells (pointed by *red arrows*), *Bv* blood vessels, *M* muscle, *black small bar* 10 µm. *Right graph* VEGF-expressing cells per 200 μm PM length in cross sections. Data were presented as mean ± SD (n = 6) and were analyzed using t test. **b** α-SMA (a myofibroblast marker) was detected using mouse monoclonal anti-α-SMA antibody from Sigma-Aldrich. *Left graph* a typical microscopic view, *Dark brown stain* α-SMA-expressing cells or myofibroblasts (pointed by *red arrows*), *Bv* blood vessels, *M* muscle, *black small bar* 10 µm. *Right graph* α-SMA-expressing cells per 200 μm PM length in cross sections. Data were presented as mean ± SD (n = 6) and were analyzed using t test. **c** Macrophages were detected using mouse monoclonal anti-MAC387 antibody from Santa Cruz Biotech. *Left graph* a typical microscopic view, *Dark brown stain* MAC387-expressing cells or macrophages (pointed by *red arrows*), *M* muscle, *black small bar* 10 µm. *Right graph* MAC387-expressing cells per 200 μm PM length in cross sections. Data were presented as mean ± SD (n = 6) and were analyzed using t test

To further understand the pathways by which PYS induced severe pathological changes in the peritoneum compared to HPG, the expression of α-SMA (a marker of myofibroblast differentiation), MAC387 (a marker of macrophages) and VEGF (a pro-angiogenic growth factor) was examined using immunohistochemical stain of peritoneal tissue sections. As shown in Fig. 4a, there were more cells stained positively with α-SMA in tissue sections of PYS group than those in HPG or Control group (15.0 ± 6.95 in PYS versus 2.71 ± 1.87 in HPG, p = 0.0019, two-tailed t test, n = 6). In addition to the positive stain of α-SMA in smooth muscle cells of the blood vessels, myofibroblasts were seen in the submesothelial layer as well as on the surface of the peritoneum in PYS group, whereas in other groups fewer of these cells were observed on the peritoneal surface. Also, there were more infiltrating macrophages in PYS than in HPG group (20.83 ± 14.03 in PYS versus 7.58 ± 3.75 in HPG, p = 0.0494, two-tailed t test, n = 6), which is consistent with more severity of peritoneal injury in PYS than that in HPG. In addition to that, the macrophages in the PYS group were localized in both inside the submesothelial layer and on its surface (Fig. 4b).

To further confirm the increase of the neoangiogenesis in the PM after chronic exposure to PYS compared to HPG, the expression of VEGF, a neoangiogenesis factor, in PM sections was examined using immunohistochemical staining. As shown in Fig. 4c, there were more VEGF-expressing cells in the submesothelial membranes in PYS group than that of HPG or Control group (58.83 ± 31.34 in PYS versus 22.8 ± 10.88 in HPG, p = 0.0307; 58.83 ± 31.34 in PYS versus 3.33 ± 0.88 in Control, p = 0.0019; two-tailed t test, n = 6 rats), which was in parallel with the most blood vessels found in the PYS group (Fig. 3c).

### Compared to PYS, HPG solution significantly induces less inflammatory signaling pathways and exclusively activated lipid metabolism-related pathway

**Fig. 5:**
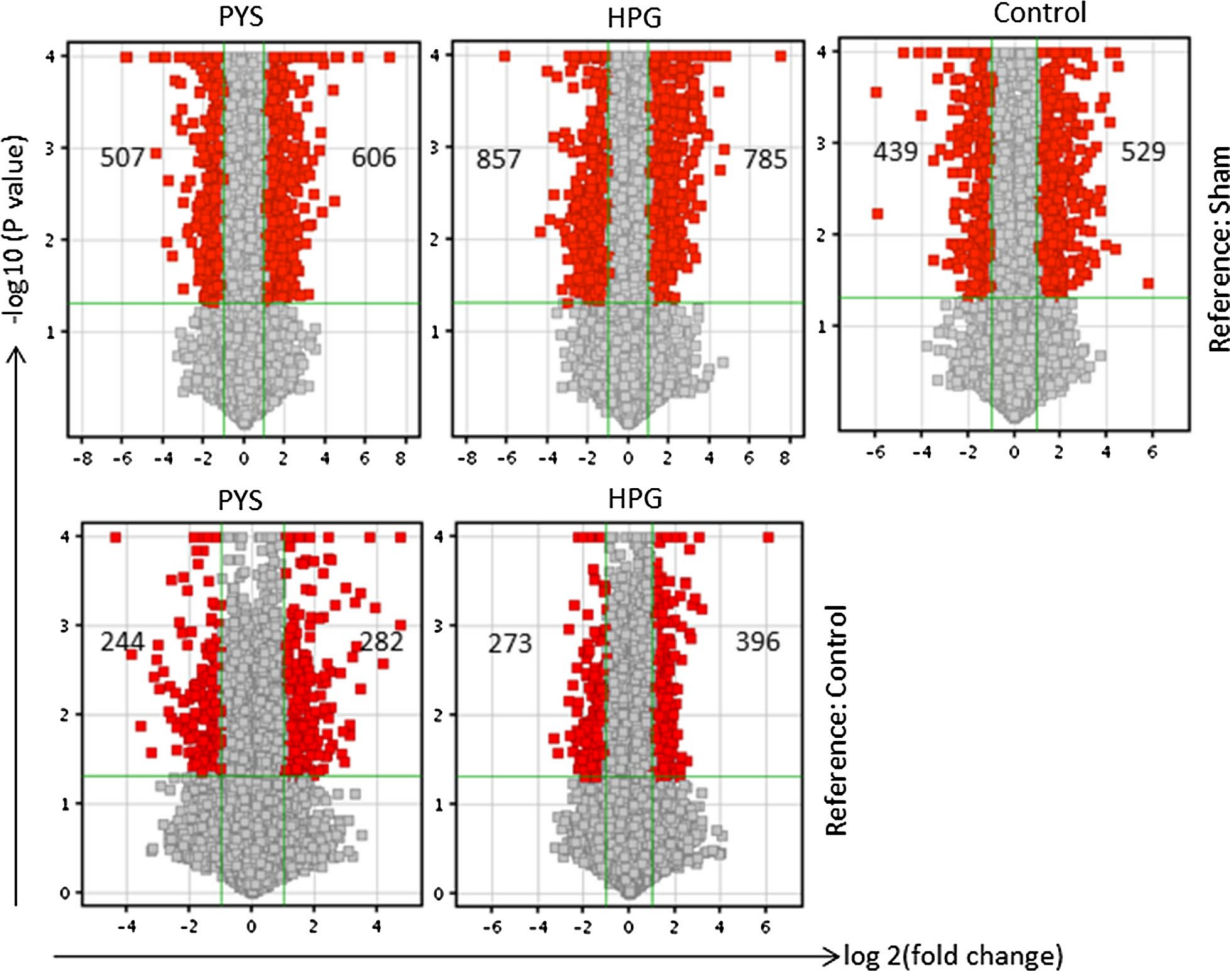
Changes in transcriptome in the PM of rats receiving daily i.p. injection of electrolyte Control, PYS or HPG solution. Venn diagram analysis of microarray data (four separate samples in each group, n = 4; one rat in Sham provided two samples) of Control, PYS or HPG was performed as compared to those of Sham or of PYS or HPG as compared to Control using the Agilent Gene Spring software. Only the transcripts that were significantly changed (*p* ≤ 0.05; FC ≥ 2.0) as compared to Sham or Control group were included and presented in this analysis. *Top panel* The changes of gene expression in Control, PYS or HPG using Sham as a reference. *Bottom panel* The change of gene expression in PYS or HPG using Control as a reference. *Positive FC* up-regulated, *negative FC* down-regulated

To further understand the molecular signaling pathways mediating the effects of daily injection of electrolyte Control, PYS or HPG solution on PM structure and function, the transcriptome-based pathway analysis was performed. Compared to Sham as a baseline there were more gene alterations in HPG group (785 up-regulated, 857 downregulated) than those in both PYS group (606 up-regulated, 507 downregulated) and Control (529 up-regulated, 439 down-regulated) (Fig. 5, upper panel). Also when HPG or PYS was compared with Control, a similar trend (HPG > PYS) was noted but with less affected gene expression (Fig. 5, bottom panel). Pathway analysis of these gene expression profilings showed that in comparison with Control, PYS or HPG with Sham, more pathways related to inflammation were significantly induced (p < 0.05) in the PM of rats receiving PYS than those in HPG or Control (Table 1), which included “Role of hypercytokinemia/hyperchemokineemia in the pathogenesis”, “IL-6 signaling”, “Toll-like signaling”, “HMGB1 signaling”, and so on. Also the p values of the pathways activated in all of three groups, such as “Granulocyte or agranulocyte adhesion and diapedesis” and “Acute phase response signaling” reached a higher statistical significance in PYS group than those in HPG or Control group (Table 1). The higher activation of the inflammatory pathways in PYS group was consistent with a significant up-regulation of several well-known pro-inflammatory mediators, such as TNF, IL-1β, CCL2 and MMP9, which were not seen in either HPG or Control group (Tables 2, 3, 4). When we compared PYS or HPG with Control, there were still more inflammatory pathways being significantly affected by glucose-based PYS than those seen in HPG group (nine in PYS versus five in HPG) (Table 5), which included “Acute phase response signaling”, “Inhibition of matrix metalloproteinase” and IL-6 signaling”. Similarly, the highly up-regulated TNF and MMP9 were found in PYS but not in HPG when Control was used as a reference in analysis (Tables 6, 7). All these data implied that TNF and/or MMP9-dependent pathways were specifically activated in the PM by chronic exposure to PYS.

**Table 1:** The *p* values (as a function) of affected inflammatory signaling pathways between Control, PYS and HPG compared to Sham

Signaling pathways (*total number of genes*)	Control	PYS	HPG
B cell development (*22*)	6.3 × 10^−6^ (+*7*)	0.001 (+*5*)	0.0002 (+*6*)
Complement (*34*)	1 × 10^−6^ (+*8*, −*1*)	1 × 10^−5^ (+*8*)	0.001 (+*7*)
Cross talk between DCs and NK cells (*68*)	0.001 (+*4*, −*6*)	0.0025 (+*4*, −*4*)	0.0016 (+*5*, −*6*)
Granulocyte adhesion and diapedesis (*145*)	0.0001 (+*6*, −*7*)	1 × 10^−11^ (+*18*, −*8*)	0.0032 (+*8*, −*5*)
Agranulocyte adhesion and diapedesis (*154*)	0.0001 (+*8*, −*7*)	1 × 10^−10^ (+*18*, +*7*)	0.004 (+*10*, −*4*)
ICOS/ICOSL signaling in Th cells (*96*)	1.3 × 10^−5^ (+*9*, −*4*)	0.0032 (+*6*, −*3*)	0.01 (+*8*, −*2*)
Antigen presentation pathway (*22*)	0.001 (+*4*)	0.0032 (+*4*)	0.01 (+*4*)
Nur 77 signaling in T cells (*43*)	5 × 10^−5^ (+*6*, −*2*)	NS (+*2*, −*2*)	0.0316 (+*4*, −*1*)
Acute phase response signaling (*157*)	0.001 (+*8*, −*4*)	1 × 10^−6^ (+*17*, −*3*)	0.0316 (+*9*, −*3*)
CD28 signaling in Th cells (*106*)	1 × 10^−5^ (+*10*, −*4*)	0.0126 (+*7*, −*2*)	0.0316 (+*7*, −*2*)
IL-17 signaling in fibroblast (*23*)	NS (−*2*)	0.0501 (+*3*, −*1*)	0.0315 (+*1*, −*3*)
Role of hypercytokinemia/hyperchemokinemia in the pathogenesis (*27*)	0.00316 (+*2*, −*1*)	3.2 × 10^−8^ (+*7*, −*2*)	NS (−*2*)
IL-10 signaling (*65*)	NS (+*2*, −*3*)	1 × 10^−5^ (+*8*, −*3*)	NS (+*1*, −*3*)
Differential regulation of cytokine production in MΦ and Th cell (*15*)	NS (+*1*, −*1*)	0.0001 (+*5*)	NS (−*1*)
Inhibition of matrix metalloproteinase (*36*)	NS (+*1*, −*1*)	0.002 (+*4*)	NS (+*4*)
TREM1 signaling (*67*)	NS (+*4*, −*1*)	0.0025 (+*7*, −*1*)	NS (+*2*, −*1*)
IL-6 signaling (*114*)	NS (+*1*, −*5*)	0.01 (+*7*, −*3*)	NS (+*1*, −*4*)
Toll-like receptor signaling (*71*)	NS (+*1*, −*1*)	0.0126 (+*6*, −*1*)	NS (−*2*)
MIF-mediated glucocortical regulation (*29*)	NS (+*1*)	0.0501 (+*4*)	NS (+*3*)
MIF regulation of innate immunity (*73*)	NS (+*1*)	0.0501 (+*4*)	NS (+*3*, −*1*)
HMGB1 signaling (*112*)	NS (+*1*, −*5*)	0.0501 (+*6*, −*2*)	NS (+*1*, −*3*)

The p value was calculated by t test as compared with the baseline sham control

*DC* dendritic cell, *NK* natural killer, *Th* T helper, *MΦ* macrophage, *ICOS* inducible costimulator, *ICOSL* inducible costimulatory ligand, *TREM1* triggering receptor expressed on myeloid cells 1, *MIF* macrophage migration inhibitory factor, *HMGB1* high mobility group box 1, *NS* not significant, *+* up-regulated number of genes, *−* down-regulated number of genes

**Table 2:** The inflammatory signaling pathways and related gene transcripts affected by daily injection of electrolyte solution (Control) compared to Sham

Signaling pathways (*p value*)	Up regulated genes	Down regulated genes
Complement (*1* × *10* ^−*6*^)	C2, C3, C6, C4BPA, CFD, ITGB2, ITGAM, ITGAX	C7
B cell development (6 × *10* ^−*6*^)	HLA-DRA, HLA-DRB5, HLA-DQB1, IL7, IL7R, PTPRC, SPN	–
CD28 signaling in Th cells (*1* × *10* ^−*5*^)	HLA-DRA, HLA-DRB5, HLA-DQB1, CD247, CD3E, CD3G, GRAP2, PAK1, PTPRC, VAV1	Calm1, FOS, PIK3C2G, PIK3R3
ICOs/ICOSL signaling in Th cells (*1.3* × *10* ^−*5*^)	CD247, CD3E, CD3G, GRAP2, HLA-DQB1, HLA-DRA, HLA-DRB5, PTPRC, VAV1	Calm1, CAMK2D, PIK3C2G, PIK3R3
Nur 77 signaling in T cells (*5* × *10* ^−*5*^)	CD247, CD3G, HLA-DRA, HLA-DRB5, HLA-DQB1, CD3E	Calm1, CASP3
Agranulocyte adhesion and diapedesis (*0.0001*)	ACTC1, AOC3, CCL4, CCL9, EZR, IL18, ITGB2, MYH3	CCL20, CD34, CLDN5, CXCL2, CXCR4, ICAM2, MMP27
Granulocyte adhesion and diapedesis (*0.00013*)	CCL4, CCL9, EZR, IL18, ITGAM, ITGB2	CCL20, CLDN5, CXCL2, CXCR4, ICAM2, IL1R2, MMP27
Cross talk between DCs and NK cells (*0.001*)	ACTG2, HLA-DRA, HLA-DRB5, IL18	IL6, MIBC, FSCN1, CAMK2D, ACTC1, TNFSF10
Antigen presentation pathway (*0.001*)	CIITA, CD74, HLA-DRA, HLA-DRB5	–
Acute phase response signaling (*0.001*)	AGT, C2, C3, C4BPA, IL18, KLKB1, RBP4, SERPINA3	FOS, IL6, PIK3R3, VWF
Role of hypercytokinemia/hyperchemokinemia in the pathogenesis (*0.003162*)	CCL4, IL18	IL6
TREM1 signaling (*0.0631*, *NS*)	CIITA, IL18, ITGAX, TLR11	IL6
IL-10 signaling (*0.0631*, *NS*)	IL18, IL10RA	FOS, IL6, IL1R2
Differential regulation of cytokine production in MΦ and Th cell (*0.1*, *NS*)	CCL4	IL6
IL-6 signaling (*0.1259*, *NS*)	IL18	IL6, FOS, IL1R2, PIK3C2G, PIK3R3
HMGB1 signaling (*0.1259*, *NS*)	IL18	IL6, FOS, PIK3C2G, PIK3R3, RHOJ
Inhibition of matrix metalloproteinase (*0.2512*, *NS*)	THBS2	MMP27
IL-17 signaling in fibroblast (*0.2512*, *NS*)		IL6, FOS
MIF regulation of innate immunity (*0.3162*, *NS*)	CD74	FOS
MIF-mediated glucocortical regulation (*0.5012*, *NS*)	CD74	–
Toll-like receptor signaling (*1.0*, *NS*)	IL18	FOS

The affected signaling pathways were ranked from the most (top) to the least significant (bottom) based on the p value

*DC* dendritic cell, *NK* natural killer, *Th* T helper, *MΦ* macrophage, *ICOS* inducible costimulator, *ICOSL* inducible costimulatory ligand, *TREM1* triggering receptor expressed on myeloid cells 1, *MIF* macrophage migration inhibitory factor, *HMGB1* high mobility group box 1, *NS* not significant

**Table 3:** The inflammatory signaling pathways and related gene transcripts affected by daily injection of PYS solution compared to Sham

Signaling pathways (*p value*)	Up regulated genes	Down regulated genes
Granulocyte adhesion and diapedesis (*1* × *10* ^−*11*^)	C5AR1, CCL2, CCL4, CCL19, CCL21, CXCL13, CXCL14, CXCR2, EZR, IL1B, IL1RN, ITGB2, MMP9, MMP16, SELL, TNF, TNFRSF11B, VCAM1	CLDN5, CXCL2, CXCR4, ICAM2, IL33, IL1RAPL1, MMP27, THY1
Agranulocyte adhesion and diapedesis (*1* × *10* ^−*10*^)	AOC3, C5AR1, CCL2, CCL4, CCL19, CCL21, CXCL13, CXCL14, CXCR2, EZR, IL1B, IL1RN, ITGB2, MMP9, MMP16, SELL, TNF, VCAM1	CD34, CLDN5, CXCL2, CXCR4, ICAM2, IL33, MMP27
Role of hypercytokinemia/hyperchemokinemia in the pathogenesis (*3* × *10* ^−*8*^)	CCL2, CCL4, CCR1, CCR5, IL1B, IL1RN, TNF	IL6, IL33
Acute phase response signaling (*1* × *10* ^−*6*^)	A2M, AGT, C3, C4BPA, CFB, FGG, HP, IKBKE, IL1B, KLKB1, RBP1, RBP4, SERPINA3, SERPINE1, TNF, TNFRSF11B	IL6, IL33, RB97
Complement (*1* × *10* ^−*5*^)	C3, C6, C4BPA, C5AR1, CFD, ITGB2, CFB, ITGAM	–
IL-10 signaling (*1* × *10* ^−*5*^)	IKBKE, CCR1, CCR5, CD14, FCGR2B, IL1B, IL1RN, TNF	IL6, IL33, IL1RAPL1
Differential regulation of cytokine production in MΦ and Th cell (*0.0001*)	CCL2, CCL4, IL1B, LCN2, TNF	–
B cell development (*0.001*)	HLA-DRA, HLA-DRB5, IL7, IL7R, PTPRC	–
Inhibition of matrix metalloproteinase (*0.002*)	A2M, MMP9, MMP16, TIMP1	MMP27, TIMP4
Cross talk between DCs and NK cells (*0.0025*)	HLA-DRA, HLA-DRB5, TNF, TLR3	IL6, IL4, FSCN1, CAMK2D
TREM1 signaling (*0.0025*)	CCL2, CIITA, FCGR2B, IL1B, NLRP3, TLR3, TNF	IL6
ICOS/ICOSL signaling in Th cells (*0.00316*)	HLA-DRA, HLA-DRB5, IKBKE, ITK, PTPRC, GRAPB2	Calm1, CAMK2D, LAT
Antigen presentation pathway (*0.00316*)	HLA-DRA, HLA-DRB5, CD74, CIITA	–
IL-6 signaling (*0.01*)	IKBKE, A2M, CD14, IL1B, IL1RN, TNF, TNFRSF11B	IL6, IL33, IL1RAPL1
CD28 signaling in Th cells (*0.0126*)	HLA-DRA, HLA-DRB5, PTPRC, PAK1, GRAP2, IKBKE, ITK	Calm1, LAT
Toll-like receptor signaling (*0.0126*)	CD14, IL1B, IL1RN, TLR3, TNF, UBD	IL33
IL-17 signaling in fibroblast (*0.0501*)	IKBKE, CCL2, LCN2	IL6
MIF-mediated glucocortical regulation (*0.0501*)	CD74, PLA2G2A, PLA2G5, CD14	–
MIF regulation of innate immunity (*0.0501*)	CD74, PLA2G2A, PLA2G5, CD14	–
HMGB1 signaling (*0.0501*)	VCAM1, TNFRSF11B, TNF, SERPINE1, IL1B, CCL2	IL6, IL4
Nur 77 signaling in T cells (*0.0631*, *NS*)	HLA-DRA, HLA-DRB5	Calm1, CASP3

The affected signaling pathways were ranked from the most (top) to the least significant (bottom) based on the p value

*DC* dendritic cell, *NK* natural killer, *Th* T helper, *MΦ* macrophage, *ICOS* inducible costimulator, *ICOSL* inducible costimulatory ligand, *TREM1* triggering receptor expressed on myeloid cells 1, *MIF* macrophage migration inhibitory factor, *HMGB1* high mobility group box 1, *NS* not significant

**Table 4:** The inflammatory signaling pathways and related gene transcripts affected by daily injection of HPG solution compared to Sham

Signaling pathways (*p value*)	Up regulated genes	Down regulated genes
B cell development (*0.00016*)	HLA-DRA, HLA-DRB5, IL7, IL7R, PTPRC, SPN	–
Complement (*0.001*)	C2, C3, C6, C4BPA, C5AR1, CFD, ITGB2	–
Cross talk between DCs and NK cells (*0.0016*)	ACTG2, HLA-DRA, HLA-DRB5, CD83	IL6, MIBC, FSCN1, FSCN2, CAMK2D, ACTG2
Granulocyte adhesion and diapedesis (*0.0032*)	C5AR1, CCL21, CXCL14, EZR, ITGA2, ITGB2, MMP11, VCAM1	CCL2, CXCL2, CXCL10, CXCR4, THY1
Agranulocyte adhesion and diapedesis (*0.004*)	ACTG2, VCAM1, EZR, ITGB2, C5AR1, ITGA2, MMP11, CXCL14, CCL21, AOC3	CCL2, CXCL2, CXCL10, CXCR4
ICOS/ICOSL signaling in Th cells (*0.01*)	CD247, CD3G, HLA-DRA, HLA-DRB5, ICOSLG, IKBKE, ITK, PTPRC	Calm1, CAMK2D
Antigen presentation pathway (*0.01*)	CIITA, CD74, HLA-DRA, HLA-DRB5	
Nur 77 signaling in T cells (*0.0316*)	CD247, CD3G, HLA-DRA, HLA-DRB5	Calm1
Acute phase response signaling (*0.0316*)	C2, SERPINA3, IKBKE, ITIH4, C3, FGG, AGT, RBP4, C4BPA	IL6, FOS, MAPK11
CD28 signaling in Th cells (*0.0316*)	HLA-DRA, HLA-DRB5, PTPRC, CD247, CD3G, IKBKE, ITK	Calm1, FOS
IL-17 signaling in fibroblast (*0.0316*)	IKBKE	IL6, FOS, MAPK11
Role of hypercytokinemia/hyperchemokinemia in the pathogenesis (*0.3162*, *NS*)	–	CCCL10, IL6
IL-10 signaling (*0.2512*, *NS*)	IKBKE	IL6, FOS, MAPK11
Differential regulation of cytokine production in MΦ and Th cells (*0.5012*, *NS*)	–	IL6
Inhibition of matrix metalloproteinase (*0.0631*, *NS*)	LRP1, MMP11, SDC1, THBS2	–
TREM1 signaling (*0.5012*, *NS*)	CD83, CIITA	IL6
IL-6 signaling (*0.5012*, *NS*)	IKBKE	IL6, FOS, MAPK11, CSNK2A2
Toll-like receptor signaling (*1.0*, *NS*)	–	FOS, MAPK11
MIF-mediated glucocortical regulation (*0.1259*, *NS*)	CD74, PLA2G2A, PLA2G5	–
MIF regulation of innate immunity (*0.0794*, *NS*)	CD74, PLA2G2A, PLA2G5	FOS
HMGB1 signaling (*1.0*, *NS*)	VCAM1	IL6, FOS, MAPK11

The affected signaling pathways were ranked from the most (top) to the least significant (bottom) based on the *p* value

*DC* dendritic cell, *NK* natural killer, *Th* T helper, *MΦ* macrophage, *ICOS* inducible costimulator, *ICOSL* inducible costimulatory ligand, *TREM1* triggering receptor expressed on myeloid cells 1, *MIF* macrophage migration inhibitory factor, *HMGB1* high mobility group box 1, *NS* not significant

**Table 5:** The *p* values (as a function) of affected inflammatory signaling pathways between PYS and HPG compared to Control

Signaling pathways (*total number of genes*)	PYS	HPG
B cell development (*35*)	NS (−*2*)	0.023 (−*3*)
Complement (*37*)	NS (+*2*)	NS (+*1*)
Cross talk between DCs and NK cells (*89*)	0.0083 (+*1*, −*4*)	NS (−*4*)
Granulocyte adhesion and diapedesis (*177*)	1.32 × 10^−6^ (+*11*, −*2*)	0.0014 (+*7*, −*3*)
Agranulocyte adhesion and diapedesis (*189*)	7.4 × 10^−5^ (+*10*, −*1*)	0.0071 (+*7*, −*2*)
ICOS/ICOSL signaling in Th cells (*122*)	NS (+2, −*2*)	0.0063 (+*3*, −*4*)
Antigen presentation pathway (*37*)	NS (−2)	NS (−*1*)
Nur 77 signaling in T cells (*59*)	NS (+*1*, −*1*)	NS (−*1*)
Acute phase response signaling (*169*)	7.8 × 10^−7^ (+*11*, −*2*)	NS (+*4*)
CD28 signaling in Th cells (*131*)	NS (+*2*, −*2*)	0.0302 (+*2*, −*4*)
IL-17A signaling in fibroblast (*35*)	0.0126 (+*1*, −*2*)	NS (−*1*)
Role of hypercytokinemia/hyperchemokinemia in the pathogenesis (*43*)	NS (+*1*)	NS (−*1*)
IL-10 signaling (*68*)	NS (+*2*, −*1*)	NC
Differential regulation of cytokine production in MΦ and Th cell (*18*)	NS (+*1*)	NC
Inhibition of matrix metalloproteinase (*39*)	0.0002 (+*3*, −*2*)	NC
TREM1 signaling (*75*)	NS (+*1*, −*1*)	NC
IL-6 signaling (*127*)	0.0021 (+*6*, −*1*)	NS (+*3*, −*1*)
Toll-like receptor signaling (*74*)	NS (+*1*, −*1*)	NC
MIF-mediated glucocortical regulation (*33*)	NS (+*1*, −*1*)	NS (+*2*)
MIF regulation of innate immunity (*41*)	0.0195 (+*2*, −*1*)	NS (+*2*)
HMGB1 signaling (*133*)	0.0389 (+*4*, −*1*)	NS (+*3*)

The p value was calculated by t test as compared with the electrolye solution control

*DC* dendritic cell, *NK* natural killer, *Th* T helper, *MΦ* macrophage, *ICOS* inducible costimulator, *ICOSL* inducible costimulatory ligand, *TREM1* triggering receptor expressed on myeloid cells 1, *MIF* macrophage migration inhibitory factor, *HMGB1* high mobility group box 1, *NS* not significant, *NC* no change, *+* up-regulated number of genes, *−* down-regulated number of genes

**Table 6:** The inflammatory signaling pathways and related gene transcripts affected by daily injection of PYS compared to Control

Signaling pathways (*p value*)	Up regulated genes	Down regulated genes
Granulocyte adhesion and diapedesis (*1.32* × *10* ^−*6*^)	SELL, VCAM1, CXCL13, CXCR2, CXCL14, CCL21, CXCL3, TNF, MMP9, CCL19, TNFRSF11B	MMP27, THY1
Agranulocyte adhesion and diapedesis (*7.4* × *10* ^−*5*^)	SELL, VCAM1, CXCL13, CXCR2, CXCL14, CCL21, CXCL3, TNF, MMP9, CCL19	MMP27
Role of hypercytokinemia/hyperchemokinemia in the pathogenesis (*0.4539*, *NS*)	TNF	–
Acute phase response signaling (*7.8* × *10* ^−*7*^)	SOCS3, HP, C3, ORM1, Saa3, CFB, TNF, A2M, RBP1, FGG, TNFRSF11B	NFKBIA, CRABP1
Complement (*0.0933*, *NS*)	C3, CFB	–
IL-10 signaling (*0.0692*, *NS*)	SOCS3, TNF	NFKBIA
Differential regulation of cytokine production in MΦ and Th cell (*0.2239*, *NS*)	TNF	–
B cell development (*0.0851*, *NS*)	–	SPN, HLA-DRA
Inhibition of matrix metalloproteinase (*0.0002*)	TIMP1, A2M, MMP9	MMP27, ADAM12,
Cross talk between DCs and NK cells (*0.0083*)	TNF	HLA-DRA, CD226, HLA-E, IL4
TREM1 signaling (*0.2818*, *NS*)	TNF	Tlr11
ICOs/ICOSL signaling in Th cells (*0.0912*, *NS*)	CAMK4, PIK3C2G	NFKBIA, HLA-DRA
Antigen presentation pathway (*0.0933*, *NS*)	–	HLA-DRA, HLA-E
IL-6 signaling (*0.0021*)	SOCS3, CSNK2A1, PIK3C2G, A2M, TNF, TNFRSF11B	NFKBIA
CD28 signaling in Th cells (*0.1117*, *NS*)	CAMK4, PIK3C2G	NFKBIA, HLA-DRA
Toll-like receptor signaling (*0.2767*, *NS*)	TNF	NFKBIA
IL-17 signaling in fibroblast (*0.0126*)	LCN2, CEBPD	NFKBIA
MIF-mediated glucocortical regulation (*0.0776*, *NS*)	PLA2G2A	NFKBIA
MIF regulation of innate immunity (*0.0195*)	NOS2, PLA2G2A	NFKBIA
HMGB1 signaling (*0.0389*)	VCAM1, PIK3C2G, TNF, TNFRSF11B	IL4
Nur 77 signaling in T cells (*0.1995*, *NS*)	CAMK4	HLA-DRA

The affected signaling pathways were ranked from the most (top) to the least significant (bottom) based on the p value

*DC* dendritic cell, *NK* natural killer, *Th* T helper, *MΦ* macrophage, *ICOS* inducible costimulator, *ICOSL* inducible costimulatory ligand, *TREM1* triggering receptor expressed on myeloid cells 1, *MIF* macrophage migration inhibitory factor, *HMGB1* high mobility group box 1, *NS* not significant

**Table 7:** The inflammatory signaling pathways and related gene transcripts affected by daily injection of HPG solution compared to Control

Signaling pathways (*p value*)	Up regulated genes	Down regulated genes
B cell development (*0.0240*)	–	CD80, HLA-DRA, HLA-DQB1
Complement (*0.3006*, *NS*)	CFD	–
Cross talk between DCs and NK cells (*0.0759*, *NS*)	–	CD80, MICB, HLADRA, ACTC1
Granulocyte adhesion and diapedesis (*0.0014*)	CLDN5, CXCR2, ITGA2, CXCL14, CCL21, CCL11, CXCL6	CXCL10, IL1RAPL1
Agranulocyte adhesion and diapedesis (*0.0071*)	CLDN5, CXCR2, ITGA2, CXCL14, CCL21, CCL11, CXCL6	CXCL10, ACTC1
ICOS/ICOSL signaling in Th cells (*0.0063*)	CD80, GRAP2, HLA-DRA, PIK3C2G, ICOSLG/LOC102723996	CD80, GRAP2, HLA-DRA, HLA-DQB1
Antigen presentation pathway (*0.4875*, *NS*)	–	HLA-DRA
Nur 77 signaling in T cells (*0.0891*, *NS*)	–	CD80, HLA-DRA, HLA-DQB1
Acute phase response signaling (*0.0813*, *NS*)	PIK3R3, VWF, KRAS, CRABP1	–
CD28 signaling in Th cells (*0.0302*)	PIK3R3, PIK3C2G	CD80, GRAP2, HLA-DRA, HLA-DQB1
IL-17 signaling in fibroblast (*0.4677*, *NS*)	–	CEBPD
Role of hypercytokinemia/hyperchemokinemia in the pathogenesis (*0.5395*, *NS*)	–	CXCL10
IL-10 signaling (*NC*)	–	–
Differential regulation of cytokine production in MΦ and Th cells (*NC*)	–	–
Inhibition of matrix metalloproteinase (*NC*)	–	–
TREM1 signaling (*NC*)	–	–
IL-6 signaling (*0.1923*, *NS*)	PIK3R3, PIK3C2G, KRAS	IL1RAPL1
Toll-like receptor signaling (*NC*)	–	–
MIF-mediated glucocortical regulation (*0.1169*, *NS*)	PLA2G5, PLA2G2A	–
MIF regulation of innate immunity (*0.1660*, *NS*)	PLA2G5, PLA2G2A	–
HMGB1 signaling (*0.4246*, *NS*)	PIK3R3, PIK3C2G, KRAS	–

The affected signaling pathways were ranked from the most (top) to the least significant (bottom) based on the *p* value

*DC* dendritic cell, *NK* natural killer, *Th* T helper, *MΦ* macrophage, *ICOS* inducible costimulator, *ICOSL* inducible costimulatory ligand, *TREM1* triggering receptor expressed on myeloid cells 1, *MIF* macrophage migration inhibitory factor, *HMGB1* high mobility group box 1, *NS* not significant, *NC* no change

In addition to the pathways mediating inflammation as listed in Table 1, the analysis of global canonical pathways showed the function of “Triacylglycerol degradation”, CDP diacylglycerol biosynthesis I”, “Phosphotidylglycerol biosynthesis II (non plastidic)” and “Triacyglycerol biosynthesis” was found only in the MP of rats receiving HPG (Table 8), which may be related to the local metabolism of this polymer.

**Table 8:** Triacylglycerol metabolism-related pathways activated by daily injection of HPG solution as compared to Sham control

Pathways (*p value*)	Symbol	Entrez gene name	Fold upregulation
Triacylglycerol degradation (*0.0158*)	FAAH	Fatty acid amide hydrolase	2.058
	LIPE	Lipase, hormone-sensitive	2.014
	LPL	Lipoprotein lipase	4.845
	PNPLA3	Patatin like phospholipase domain containing 3	2.602
CDP diacylglycerol biosynthesis I (*0.0316*)	AGPAT2	1-acylglycerol-3-phosphate O-acyltransferase 2	3.65
	CDS1	CDP-diacylglycerol synthase 1	2.755
	GPAT3	Glycerol-3-phosphate acyltransferase 3	2.103
Phosphotidylglycerol biosynthesis II (non plastidic) (*0.0398*)	AGPAT2	1-acylglycerol-3-phosphate O-acyltransferase 2	3.65
	CDS1	CDP-diacylglycerol synthase 1	2.755
	GPAT3	Glycerol-3-phosphate acyltransferase 3	2.103
Triacyglycerol biosynthesis (*0.0398*)	AGPAT2	1-acylglycerol-3-phosphate O-acyltransferase 2	3.65
	DGAT2	Diacylglycerol O-acyltransferase 2	3.914
	GPAT3	Glycerol-3-phosphate acyltransferase 3	2.103
	LPIN2	Lipin 2	2.193

## Discussion

Our previous studies have demonstrated the efficacy of small HPG molecules (0.5–3 kDa) as potential osmotic agents in fluid removal and their biocompatibility with the peritoneum in a rat model of acute (4 h) PD [18, 19]. The present study has compared the long-term effects of HPG (1 kDa) with glucose on overall health status and its local influence on the peritoneum using a rat model of chronic PD, and has demonstrated that as compared to electrolyte solution, HPG did not induce any change in the blood chemistry, but IP injection of PYS significantly changed the levels of Cre, ALP, ALB and Glob (including ALB/Glob ratio) during 3 months of treatment. These data suggest that the long-term effects of HPG on the glucose metabolism, electrolytes, liver and kidney function were not different from those of Control, whereas the use of PYS may associate with the dysfunction of the kidney and/or chronic immune activation or inflammation as PYS-treated rats have the elevated levels of serum Cre, ALP and Glob and a decrease in ALB or ALB to Glob ratio. Interestingly, hypoalbuminemia is of concern in the disease management of PD patients [25], suggesting that the use of HPG-based PD solution may improve this condition. Locally, although there were some tissue damage and functional impairment in the PM in rats after 3 months of daily exposure to hyperosmotic HPG solution compared to hypoosmotic electrolyte solution, the difference between these two groups was not significant with this limited number of animals. Unlike HPG solution, PYS induced a significant change in the structure of PM and attenuates its UF capacity, which is associated with increases in angiogenesis or VEGF expression, myofibroblast population, macrophage infiltration and activation of inflammatory pathways, especially the TNF and/or MMP9-dependent pathways as indicated by the transcriptome-based analysis.

In PD, glucose has been used as the most common osmotic agent for several decades to achieve adequate fluid removal. Because of a high level of glucose, and mostly glucose degradation products in the PD solution (e.g. PYS) that can lead to peritoneal membrane damage, fibrosis development and eventually ultrafiltration failure [7, 11, 12], alternative osmotic agents, such as polyglucose (icodextrin, Baxter Extraneal) or amino acids (Baxter Nutrineal) are developed, and their efficiency in preserving the peritoneal membrane integrity and function has been documented in both experimental and clinical studies [20, 26]. However, both icodextrin and amino acid therapies have several drawbacks that could limit their broad therapeutic uptake. For example, amino acid PD therapy has been used in clinical trials to replace only one glucose-based PD bag per day; in the mainstream clinic, it is typically reserved for PD patients with significant cachexia/hypoalbuminemia. Also, amino acids are small molecules and therefore are weak osmotic agents, and they do cause issues with metabolic acidosis and increase nitrogen loading. Icodextrin is a well-established glucose polymer only used for the long dwell in a portion of PD patients mostly in developed countries, and it is currently not approved for twice daily use and has slow fluid kinetics which limits its utility in short dwell or CAPD exchange. All these drawbacks in the use of these alternative osmotic agents may require us to develop a new osmotic agent.

Chronic injury of the PM in response to glucose-based PD solution triggers a cascade of chronic inflammation, tissue repair and remodeling (e.g. tissue fibrosis, angiogenesis) [27, 28], which are associated with the presence of persistent cellular infiltration (e.g. macrophages and lymphocytes), inflammatory and anti-inflammatory factors (e.g. TNF-α, IL-6, TGF-β) and tissue growth factors (e.g. VEGF, TGF-β) [11, 28–32]. In the comparison of HPG with PYS group, our data showed that in PYS group there was more damage in the structure of the PM as represented as PM thickness; this was positively correlated with an increase in infiltrating macrophages and more myofibroblasts, which may suggest that an increase in infiltrating macrophages is a part of chronic inflammation cascade activated by PYS, and the mesothelial cells in the inflammatory peritoneum may be more prone to differentiate to the myofibroblasts. Moreover, VEGF is a key factor for the neoangiogenesis, and can be produced by many types of cells including peritoneal mesothelial cells in response to pro-inflammatory cytokine stimulation [33] or a high-glucose PD solution [34]. We did observe an increased expression of VEGF with immunohistochemistry that correlates with an increase in neoangiogenesis (capillary density) within the PM in the PYS group; this was associated with increased loss of UF capacity (Fig. 2). In both experimental and clinical studies, PM fibrosis and neoangiogenesis are the two most important factors closely associated with UFF [35].

Furthermore, our transcriptome-based analysis confirmed that many inflammatory pathways were activated in the PM of rats receiving daily i.p. injection of either HPG or PYS. However, the number of inflammatory pathways and degree of statistical significance was greater in the PYS than HPG group compared to Sham (Table 1) or to Control (Table 5). These findings provide strong biological connection between the gene expression profiles of the PM after exposure to a variety of PD solutions and the morphological and functional alterations observed in our experiments. Furthermore, one interesting finding from this microarray analysis was the upregulation within the PM of triacylglycerol metabolism-related pathways with HPG exposure (Table 8), indicating that HPG can be probably degraded in part locally by mesothelial cells and macrophages in the peritoneal membrane, which however remains further elusive.

One has to acknowledge that there are several limitations of this study in term of scale and clinical relevance. Firstly, this was a small scale of experimental study, which was designed to examine the long-term impact of HPG on PM structure and function, but not on other organs or systems, such as the kidney, liver and circulatory system. Secondly, because of the species differences (rats versus humans) in the genomes, body size and anatomy, organ/tissue structure and function and metabolism or physiology, the human reactions to HPG versus PYS, particularly the activation of pathways, may not be the same as we saw in the rats in this study. Further studies using human subjects will be needed to verify these data. Finally, a deficiency of methodologies for this experimental study, such as the limited number of animals in each group, and IP injection of a PD solution to the rats versus using a catheter in patients, may lead to different conclusions.

## Conclusion

Our data in this study demonstrates the superiority of replacing glucose with HPG on the preservation of PM structure and function after daily exposure for 3 months in this experimental model of chronic PD. This study also conclusively links the deleterious effects of glucose to the alterations of inflammatory gene pathways in the PM, and it appears HPG may be effective in mitigating some of these problems. Taken together with our series of acute experiments with HPG-based PD solutions [18, 19], our present study clearly suggests that HPG is a promising novel osmotic agent for use in PD. Currently we are investigating the metabolic and pharmacokinetic pathways of HPG in order to ensure that our novel solution is safe for long-term use in patients with compromised renal function.

## Additional file

**Additional file 1: Table S1.** The blood chemistry of Wistar rats during 3 months of once-daily intraperitoneal injection of PD solutions.

## Abbreviations

ALP: alkaline phosphatase
ALT: alanine aminotransferase
ANOVA: analysis of variance
BUN: urea nitrogen
BWG: bodyweight gain
H&E: hematoxylin and eosin
HPG: hyperbranched polyglycerol
IP: intraperitoneal injection
IPA: ingenuity pathway analyses
PD: peritoneal dialysis
PM: peritoneal membrane
SD: standard derivation
SMA: smooth muscle actin
TBIL: total bilirubin
TP: total protein
UFF: ultrafiltration failure
VEGF: vascular endothelial growth factor
