# Supplementary material for: Hyperbranched polyglycerol is superior to glucose for long-term preservation of peritoneal membrane in a rat model of chronic peritoneal dialysis
Source: J Transl Med. 2016 Dec 13;14:338. doi: 10.1186/s12967-016-1098-z (PMC5153908; doi:10.1186/s12967-016-1098-z)
Supplement: Supplementary file 1 — Additional file 1: Table S1. The blood chemistry of Wistar rats during 3 months of once-daily intraperitoneal injection of PD solutions. [file 12967_2016_1098_MOESM1_ESM.docx]

Additional file 1: Table S1. The blood chemistry of Wistar rats during 3 months of once-daily intraperitoneal injection of PD solutions

| Name and its initial level (n = 3) | Mths | Sham (n = 3) | Control (n = 7) | PYS (n = 7) | HPG (n = 8) | PYS vs. Control | HPG vs. Control | PYS vs. HPG |
| --- | --- | --- | --- | --- | --- | --- | --- | --- |
| ALB (g/L):  42 ± 1 | 1 | 45.67 ± 0.58 | 46.43 ± 2.99 | 44.43 ± 2.57 | 45.13 ± 3.0 | 0.003 | 0.7796 | 0.0403 |
|  | 2 | 49.67 ± 1.53 | 45.14 ± 3.13 | 44.0 ± 2.94 | 46.0 ± 5.07 |  |  |  |
|  | 3 | 51.0 ± 1.0 | 47.29 ± 2.22 | 42.0 ± 3.22 | 46.75 ± 5.37 |  |  |  |
| ALP (U/L):  228 ± 8.19 | 1 | 178.67 ± 23.44 | 172.57 ± 52.2 | 252.43 ± 96.58 | 168.5 ± 55.29 | 0.0074 | 0.4325 | 0.0204 |
|  | 2 | 193.0 ± 28.79 | 177.86 ± 38.6 | 237.28 ± 94.49 | 194.13 ± 39.36 |  |  |  |
|  | 3 | 163.0 ± 59.57 | 183.14 ± 31.86 | 238.14 ± 98.36 | 203.75 ± 52.67 |  |  |  |
| ALT (U/L):  50 ± 4.36 | 1 | 51.0 ± 4.36 | 50.0 ± 7.35 | 50.71 ± 6.18 | 53.13 ± 18.26 | 0.2272 | 0.3293 | 0.1295 |
|  | 2 | 69.67 ± 3.79 | 59.14 ± 11.35 | 51.71 ± 7.43 | 66.25 ± 33.42 |  |  |  |
|  | 3 | 72.67 ± 9.73 | 61.43 ± 6.19 | 57.0 ± 16.19 | 69.63 ± 27.84 |  |  |  |
| AMY (U/L):  847.67 ± 89.58 | 1 | 897.0 ± 49.69 | 902.71 ± 50.23 | 913.57 ± 80.73 | 813.13 ± 106.5 | 0.6977 | 0.0747 | 0.0765 |
|  | 2 | 947.33 ± 60.07 | 905.29 ± 38.42 | 934.0 ± 120.39 | 842.88 ± 104.8 |  |  |  |
|  | 3 | 914.0 ± 101.3 | 861.14 ± 63.83 | 851.43 ± 107.8 | 864.25 ± 128.8 |  |  |  |
| BUN(mmol/L):  6.43 ± 1.27 | 1 | 6.97 ± 1.33 | 6.71 ± 0.82 | 7.2 ± 0.44 | 6.53 ± 0.73 | 0.7100 | 0.3706 | 0.4555 |
|  | 2 | 7.9 ± 0.78 | 7.43 ± 1.3 | 6.8 ± 0.52 | 6.95 ± 0.67 |  |  |  |
|  | 3 | 7.33 ± 1.01 | 6.91 ± 0.57 | 6.79 ± 0.49 | 6.93 ± 0.42 |  |  |  |
| Ca^2+^ (mmol/L);  2.72 ± 0.04 | 1 | 2.85 ± 0.04 | 2.85 ± 0.05 | 2.85 ± 0.12 | 2.64 ± 0.3 | 0.7384 | 0.1608 | 0.2518 |
|  | 2 | 2.79 ± 0.08 | 2.75 ± 0.11 | 2.78 ± 0.13 | 2.79 ± 0.09 |  |  |  |
|  | 3 | 2.74 ± 0.07 | 2.77 ± 0.09 | 2.71 ± 0.04 | 2.75 ± 0.07 |  |  |  |
| Cre (µmol/L):  19 ± 3.46 | 1 | 21.67 ± 8.08 | 20.86 ± 5.79 | 19.43 ± 3.99 | 20.38 ± 6.3 | 0.0231 | 0.8439 | 0.0269 |
|  | 2 | 19.67 ± 3.06 | 19.71 ± 4.46 | 27.0 ± 8.27 | 24.29 ± 6.95 |  |  |  |
|  | 3 | 22.67 ± 5.51 | 28.71 ± 8.04 | 37.29 ± 7.54 | 25.71 ± 4.92 |  |  |  |
| Glob (g/L):  20 ± 2.65 | 1 | 20.33 ± 2.52 | 25.14 ± 2.97 | 29.0 ± 2.16 | 23.75 ± 3.01 | 0.0141 | 0.7534 | 0.0171 |
|  | 2 | 20.0 ± 2.0 | 28.43 ± 4.72 | 31.14 ± 5.98 | 28.5 ± 5.73 |  |  |  |
|  | 3 | 17.0 ± 2.65 | 25.57 ± 2.3 | 28.43 ± 4.08 | 25.63 ± 6.07 |  |  |  |
| Glu (mmol/L):  7.57 ± 0.74 | 1 | 7.23 ± 1.03 | 6.19 ± 1.27 | 5.6 ± 1.58 | 6.25 ± 0.89 | 0.5669 | 0.3459 | 0.8449 |
|  | 2 | 6.7 ± 0.78 | 6.93 ± 0.5 | 6.09 ± 1.6 | 6.19 ± 1.15 |  |  |  |
|  | 3 | 7.63 ± 1.6 | 6.84 ± 1.38 | 7.59 ± 0.93 | 6.63 ± 0.83 |  |  |  |
| K^+^ (mmol/L):  6.4 ± 0.36 | 1 | 6.33 ± 0.23 | 5.76 ± 0.63 | 6.31 ± 0.42 | 6.48 ± 1.13 | 0.3172 | 0.9253 | 0.3122 |
|  | 2 | 6.2 ± 0.5 | 7.1 ± 1.22 | 6.67 ± 1.15 | 7.1 ± 0.99 |  |  |  |
|  | 3 | 5.57 ± 1.01 | 6.97 ± 0.95 | 6.04 ± 0.42 | 6.34 ± 1.29 |  |  |  |
| Na^+^ (mmol/L):  135 ± 1 | 1 | 138.33 ± 2.52 | 139.71 ± 1.98 | 140.29 ± 2.22 | 138.5 ± 4.99 | 0.9492 | 0.4573 | 0.4832 |
|  | 2 | 139.67 ± 0.58 | 140.57 ± 1.99 | 142.14 ± 4.14 | 141.38 ± 1.85 |  |  |  |
|  | 3 | 143 ± 1.73 | 143.86 ± 2.04 | 141.86 ± 2.04 | 142.5 ± 1.2 |  |  |  |
| Phos (mmol/L):  2.74 ± 0.12 | 1 | 2.3 ± 0.09 | 2.4 ± 0.23 | 2.46 ± 0.14 | 2.38 ± 0.33 | 0.0742 | 0.3350 | 0.5075 |
|  | 2 | 2.33 ± 0.26 | 2.53 ± 0.12 | 2.25 ± 0.19 | 2.41 ± 0.22 |  |  |  |
|  | 3 | 1.89 ± 0.13 | 2.33 ± 0.25 | 2.22 ± 0.2 | 2.27 ± 0.15 |  |  |  |
| TBIL (µmol/L):  5 ± 0 | 1 | 5 ± 0 | 5 ± 0 | 4.86 ± 0.38 | 4.63 ± 0.52 | 0.7167 | 0.4171 | 0.2468 |
|  | 2 | 6 ± 0 | 5.31 ± 0.79 | 5.43 ± 0.79 | 4.88 ± 0.99 |  |  |  |
|  | 3 | 7 ± 0 | 5.57 ± 1.13 | 5.86 ± 1.07 | 5.75 ± 1.04 |  |  |  |
| TP (g/L):  62.67 ± 0.57 | 1 | 66 ± 2 | 71.43 ± 3.51 | 73.57 ± 3.55 | 67.63 ± 7 | 0.7200 | 0.4108 | 0.3089 |
|  | 2 | 70.67 ± 2.08 | 73.71 ± 4.31 | 75 ± 7.64 | 74.5 ± 3.86 |  |  |  |
|  | 3 | 68 ± 1.73 | 72.71 ± 2.98 | 70.57 ± 1.51 | 72.5 ± 2.45 |  |  |  |
| ALB/Glob ratio: 2.13 ± 0.3 | 1 | 2.27 ± 0.3 | 1.87 ± 0.26 | 1.54 ± 0.14 | 1.93 ± 0.27 | 0.0022 | 0.5636 | 0.0114 |
|  | 2 | 2.51 ± 0.33 | 1.64 ± 0.37 | 1.45 ± 0.26 | 1.72 ± 0.61 |  |  |  |
|  | 3 | 3.05 ± 0.5 | 1.87 ± 0.21 | 1.52 ± 0.37 | 1.97 ± 0.72 |  |  |  |

ALB: albumin, ALP: alkaline phosphatase, ALT: alanine aminotransferase, AMY: amylase, BUN: blood urea nitrogen, Cre: creatinine, Glob: globulin, Glu: glucose, Phos: phosphate, TBIL: total bilirubin, TP: total protein. Two-way ANOVA was used to analyze the difference of two groups at different time points.
